# Fabrication of Fe–Co Magnetostrictive Fiber Reinforced Plastic Composites and Their Sensor Performance Evaluation

**DOI:** 10.3390/ma11030406

**Published:** 2018-03-09

**Authors:** Kenichi Katabira, Yu Yoshida, Atsuji Masuda, Akihito Watanabe, Fumio Narita

**Affiliations:** 1 Department of Materials Processing, Graduate School of Engineering, Tohoku University, Aoba-yama 6-6-02, Sendai 980-8579, Japan; kenichi.katabira.s2@dc.tohoku.ac.jp (K.K.); yu.yoshida.p8@dc.tohoku.ac.jp (Y.Y.); 2 Industrial Technology Center of Fukui Prefecture, 10 Kitainada, 61 Kawaiwashiduka-cho, Fukui 910-0102, Japan; a_masuda@fklab.fukui.fukui.jp; 3 Sakase Adtech Co., Ltd., 14-10 Shimoyasuda, Maruoka-cho, Sakai 910-03630, Japan; a-watanabe@sakase.co.jp

**Keywords:** composite design, Fe–Co fiber, magnetostrictive composites, inverse magnetostriction, energy harvesting

## Abstract

The inverse magnetostrictive effect is an effective property for energy harvesting; the material needs to have large magnetostriction and ease of mass production. Fe–Co alloys being magnetostrictive materials have favorable characteristics which are high strength, ductility, and excellent workability, allowing easy fabrication of Fe–Co alloy fibers. In this study, we fabricated magnetostrictive polymer composites, in which Fe–Co fibers were woven into polyester fabric, and discussed their sensor performance. Compression and bending tests were carried out to measure the magnetic flux density change, and the effects of magnetization, bias magnetic field, and the location of the fibers on the performance were discussed. It was shown that magnetic flux density change due to compression and bending is related to the magnetization of the Fe–Co fiber and the bias magnetic field. The magnetic flux density change of Fe–Co fiber reinforced plastics was larger than that of the plastics with Terfenol-D particles.

## 1. Introduction

The Internet of Things (IoT) is expected to reform our lifestyles, because it has the ability to improve sensing, actuation, communications, and control, and to create knowledge from big data [1]. On the other hand, it is desirable for IoT systems to have renewable energy sources, with a self-sustaining and maintenance-free energy harvesting ability [2]. In particular, piezoelectric ceramics/polymers, magnetostrictive alloys, and magnetoelectric composite materials have attracted attention as favorable materials for energy harvesting devices [3].

The inverse magnetostrictive effect is an effective property for energy harvesting; the materials need to have large magnetostriction and ease of mass production [4]. Terfenol-D has been an important magnetostrictive material and is widely known due to its giant magnetostriction and low magnetic anisotropy [5]. Davino et al. [6] investigated the magnetostrictive and magnetic properties of Terfenol-D subjected to several stresses and magnetic fields, and they showed that the magnetoelastic constants reach their largest values at lower compressive stresses and magnetic fields. Mori et al. [7] numerically and experimentally revealed the energy harvesting properties and behavior of Terfenol-D cantilevers with resonant tuning during dynamic bending. Terfenol-D has received much attention because of its advantages as an energy harvesting material, although its brittleness and eddy currents have prevented it from being adopted over piezoelectric materials. Therefore, a lot of researchers have applied it to composite materials. Duenas et al. [8] showed that Terfenol-D/polymer magnetostrictive composites can solve many of the disadvantages; the magnetostrictive properties of various composites have been investigated [9,10,11,12]. Furthermore, Kubicka et al. [13,14] indicated that Terfenol-D particle characteristics affect how the magnetic flux changes when under stress for Terfenol-D/epoxy composites, and Yoffe et al. [15] discussed a new process for modifying the magnetic field that is induced when an external load is applied to Terfenol-D/epoxy composites. In recent years, the suitability of epoxy-based Terfenol-D composites for sensing stress sensors has been investigated [16]. Moreover, wireless thin-layer force sensors based on washers made of Terfenol-D/epoxy composite materials have been demonstrated [17]. Galfenol, composed of iron and gallium, exhibits high magnetostriction, and some researchers have studied its characteristics as an energy harvester [18,19]; however, some drawbacks have been displayed, like difficulty in production.

Fe–Co alloys, being magnetostrictive materials, are also suitable for energy harvesting applications due to rich elements and lower cost compared with Terfenol-D and Galfenol. Recently, Yamaura et al. [20] found that alloy composition and thermomechanical treatment affects the magnetostriction of Fe_1−x_Co_x_ alloys (where x is 50–90 at %) that are processed by forging and subsequent cold rolling. Moreover, Kimura et al. [21] introduced a heat treatment for rapidly solidified Fe–Co alloys with high Co content. Fe–Co alloys have many favorable characteristics that allow easy fabrication of Fe–Co alloy fibers: high strength, ductility, and excellent workability [22]. These characteristics enable us to develop novel magnetostrictive composites [3]. The magnetostrictive continuous fibers with high aspect ratios exhibit some additional advantages, including reduced effects of demagnetization fields and strong magnetocrystalline anisotropy [23]. Consequently, it is expected that the embedding of Fe–Co fibers in a polymer matrix will result in a large magnetostriction in the composite material. Furthermore, the fact that polymer composites are lightweight and have flexible processing characteristics enables the design of high-quality composite materials, thus taking advantage of the favorable characteristics of the Fe–Co fibers.

In this study, we attempted to develop magnetostrictive polymer composites, in which Fe–Co fibers are woven into the polyester fabric. We fabricated two types of samples. Compression and bending tests were then carried out to measure the magnetic flux density change, and the effects of initial magnetization, bias magnetic field, and the location of the fibers on the performance are discussed in detail.

## 2. Materials and Methods

**Scheme 1:**
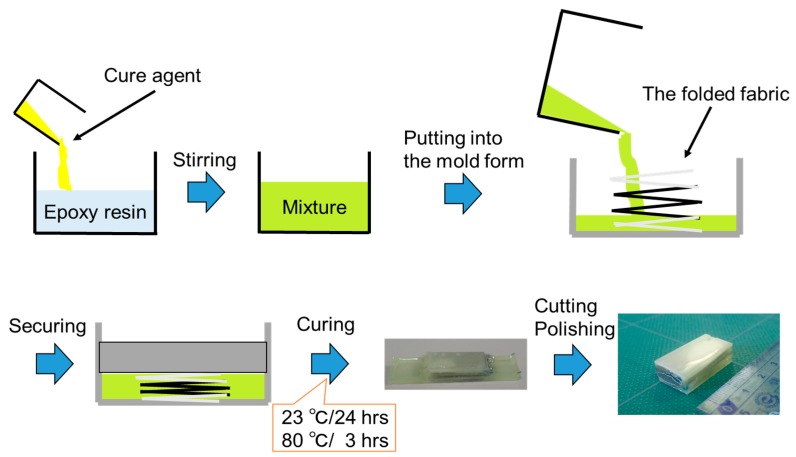
Scheme of sample fabricating procedure.

**Figure 1:**
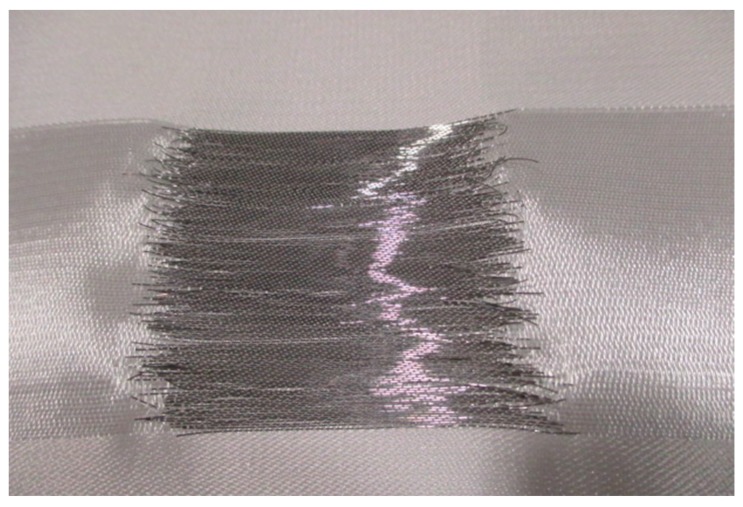
Fabric made of Fe–Co and polyester fibers.

**Figure 2:**
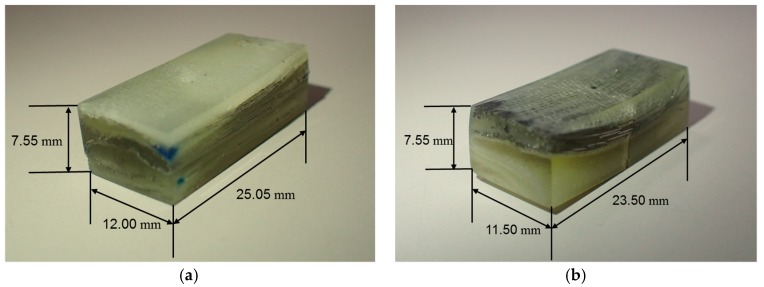
Optical images of (**a**) Type 1 specimen and (**b**) Type 2 specimen.

The procedure for fabricating the composite materials is shown in Scheme 1. The polymer matrix was made from epoxy resin based on diglycidyl ether of Bisphenol-F and a polyamine curing agent. The mixing ratio by weight was 100:55. The Fe–Co continuous fibers with a diameter of 0.2 mm were prepared by drawing. They have a columnar structure, and the easy axis in the fibers is along the <100> direction. In order to form Fe–Co fiber reinforced plastics, fabric was made by weaving with the warp of polyester fibers and the weft of polyester fibers and Fe–Co fibers, and is shown in Figure 1. The fabric was cut, folded, and put into a mold so that the easy axis in the fiber corresponded to the length direction of the mold. The stirred mixture was poured into the mold and cured for one day at room temperature, followed by curing for 3 h at 80 °C inside an oven. The optical images of the specimens are presented in Figure 2, and their final dimensions and fabric distribution are listed in Table 1. In the Type 1 specimen, Fe–Co fibers were distributed near the center of the composite material. On the other hand, in the Type 2 specimen, the fibers were distributed in one side of the composite material.

**Figure 3:**
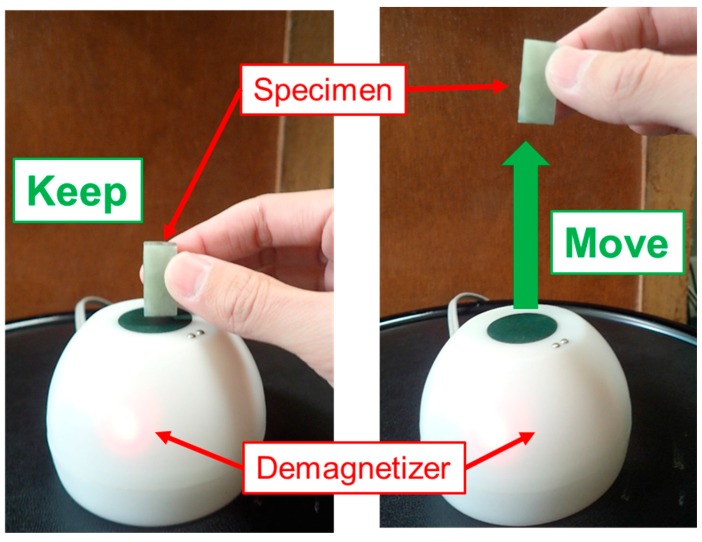
Demagnetizer.

**Figure 4:**
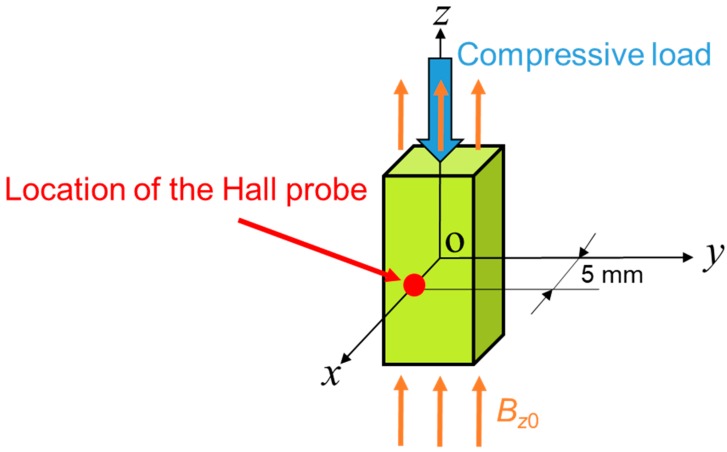
Experimental setup and location of Hall probe on compression test.

**Figure 5:**
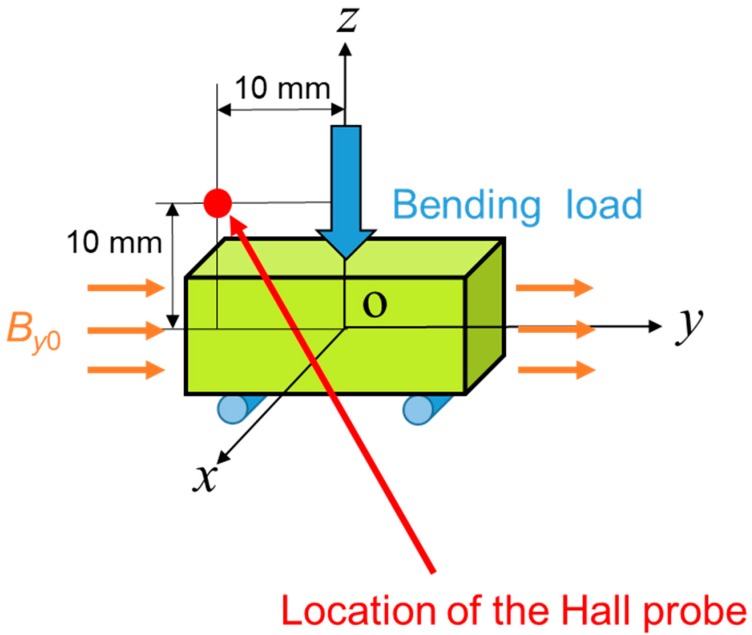
Experimental setup and location of Hall probe on three-point bending test.

The specimens were magnetized and demagnetized by using the demagnetizer as shown in Figure 3. While the demagnetizer was operating, the specimen was magnetized when kept close, and it was demagnetized when moved farther away from the demagnetizer. Here, the specimen was magnetized only in length direction of Fe–Co fiber in the samples due to its strong magnetocrystalline anisotropy [23]. Mechanical compression and three-point bending tests were carried out by using Autograph (AG-50kNXD, SHIMAZU CORPORATION, Kyoto, Japan). Figure 4 and Figure 5 show the experimental setup of compression and three-point bending tests, respectively. Let us now take the rectangular Cartesian coordinates (*x*, *y*, *z*) and the origin of the coordinate system is at the center of the sample. The compression test was conducted on a Type 1 specimen. The initial magnetization *M_z_*_0_ were approximately 1.0, 5.1, and 5.2 mT. Permanent magnets of dimensions 10 mm diameter by 5 or 10 mm length were prepared. Bias magnetic fields were controlled by different magnets, and were *Bz*_0_ = 0, 410, and 517 mT. The direction of bias magnetic field was also parallel to the length direction of Fe–Co fibers in the sample. The stress rate was 0.08 MPa/s. The magnetization vector is aligned along with the direction of the bias magnetic field, and the direction of the initial magnetization can hardly rotate with the stress. Tesla meter was used for measurement of magnetic flux density change ∆*B_z_*, which is increment or decrement of magnetic flux density. The negative sign for magnetic flux density change is omitted for convenience. Location of the Hall probe was *x* = 5 mm, *y* = 0 mm, *z* = 0 mm as shown in Figure 4. The three-point bending test was performed on Type 1 and Type 2 specimens. Bias magnetic fields *By*_0_ and the stress rate were applied in the same way as for compression tests. The initial magnetization of the Type 1 specimen were *M_y_*_0_ = 1.0 and 6.9 mT, and those of Type 2 specimen were *M_y_*_0_ = 1.0, 7.0 and 7.7 mT. The Hall probe of Tesla meter was put on *x* = 0 mm, *y* = 10 mm, *z* = 10 mm as shown in Figure 5. At this point, magnetic flux density change ∆*B_y_* was measured.

## 3. Results

**Figure 6:**
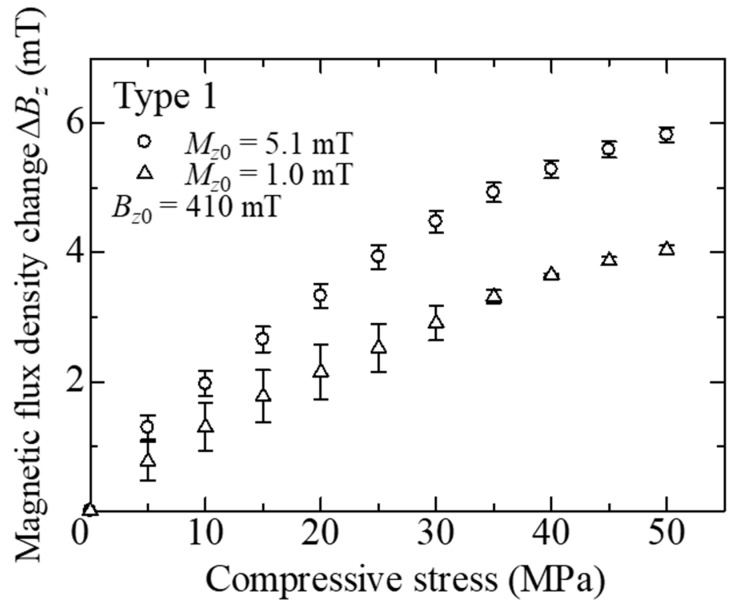
Magnetic flux density change ∆*B_z_* versus compressive stress of the Type 1 sample.

**Figure 7:**
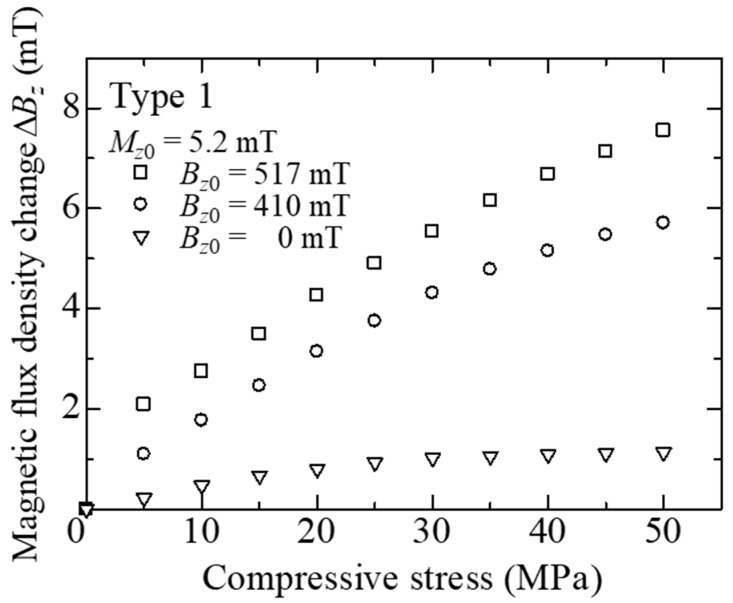
Magnetic flux density change ∆*B_z_* versus compressive stress of the Type 1 sample under various bias magnetic fields *Bz*_0_.

Here, the results of the compression test are discussed in detail. Figure 6 shows the magnetic flux density change versus compressive stress of the Type 1 sample under a bias magnetic field of *Bz*_0_ = 410 mT. The magnetic flux density change ∆*B_z_* increases with increasing compressive stress. The magnetized Fe–Co fiber shows a larger magnetic flux density change than the demagnetized one. Figure 7 shows the magnetic flux density change ∆*B_z_* versus compressive stress of the Type 1 sample under *Bz*_0_ = 0, 410, and 517 mT. The initial magnetization was approximately *M_z_*_0_ = 5.2 mT. The bias magnetic field increases the magnetic flux density change.

**Figure 8:**
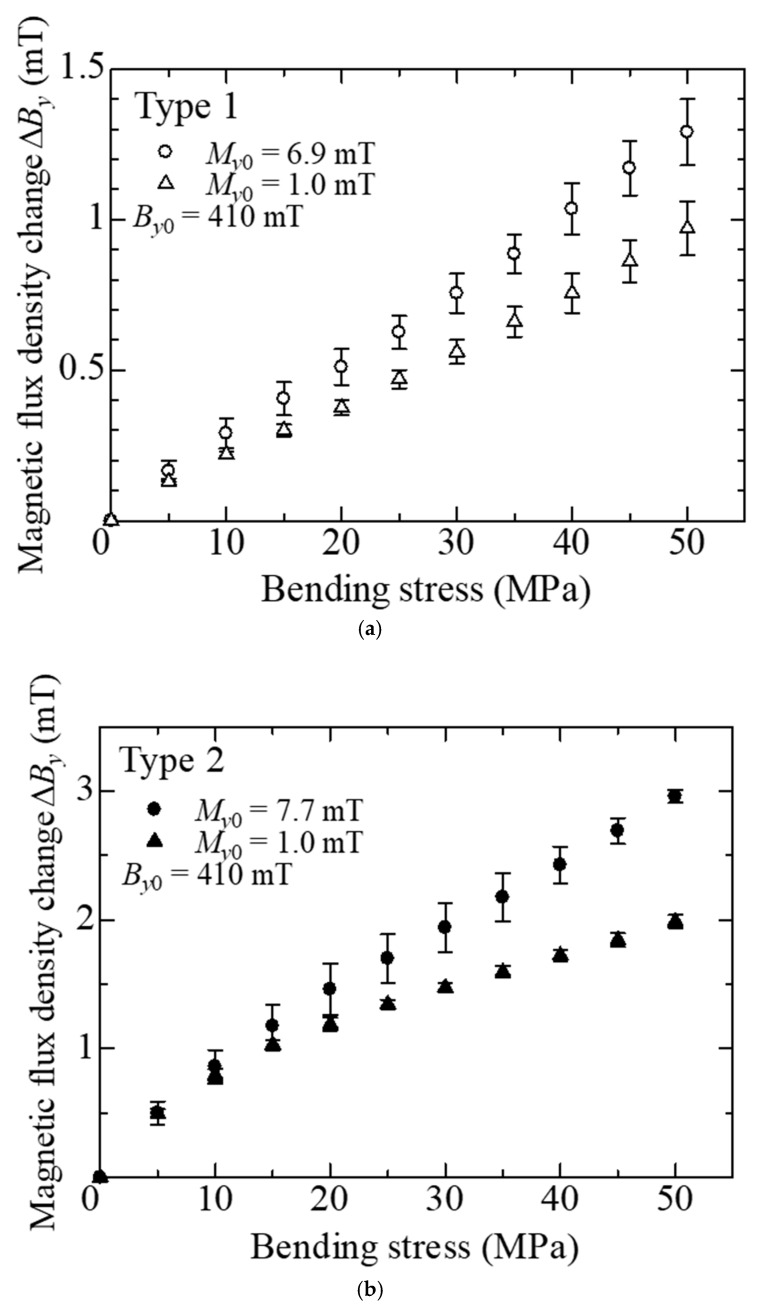
Magnetic flux density change ∆*B_y_* versus bending stress of the (**a**) Type 1 and (**b**) Type 2 samples.

**Figure 9:**
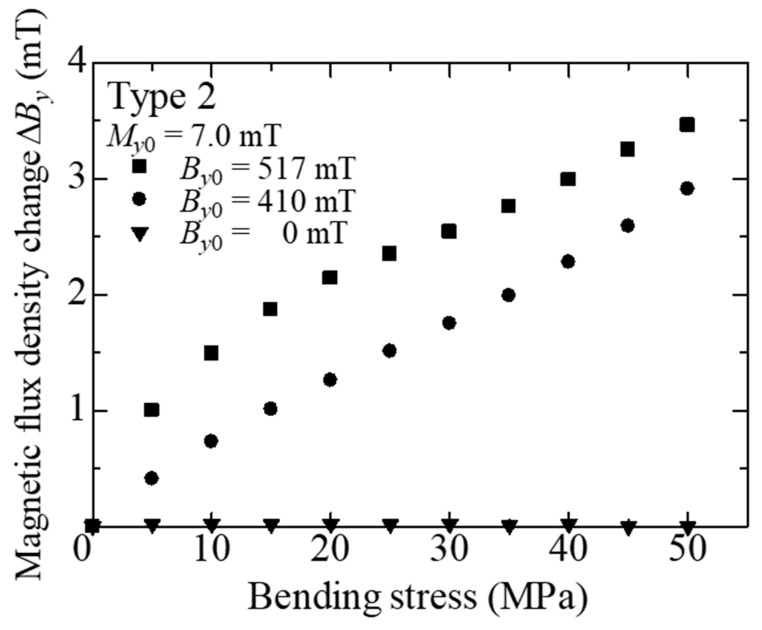
Magnetic flux density change ∆*B_y_* versus bending stress of Type 2 sample under various bias magnetic fields *B_y_*_0_.

Next, the results of three-point bending test are discussed. Figure 8 shows the dependence of the initial magnetization *M_y_*_0_ on the magnetic flux density change ∆*B_y_* versus bending stress of the Type 1 and 2 samples under *By*_0_ = 410 mT. Both magnetized specimens showed a large magnetic flux density change due to bending stress. The change of the Type 2 sample is larger than that of Type 1 sample. This is due to the location of the fibers. In the three-point bending test, the fibers of the Type 2 sample were located on the upper side of the specimen. Hence, the compressive bending stresses were applied to the fibers, and the rotation of the magnetic domain in the fibers was greater than that in the Type 1 sample. Yang et al. [24] have reported a similar phenomenon in Fe–Co-clad steel plate. Figure 9 shows the magnetic flux density change versus bending stress of the Type 2 sample under various bias magnetic fields. Magnetic flux density change increases with increasing magnetic field bias.

**Figure 10:**
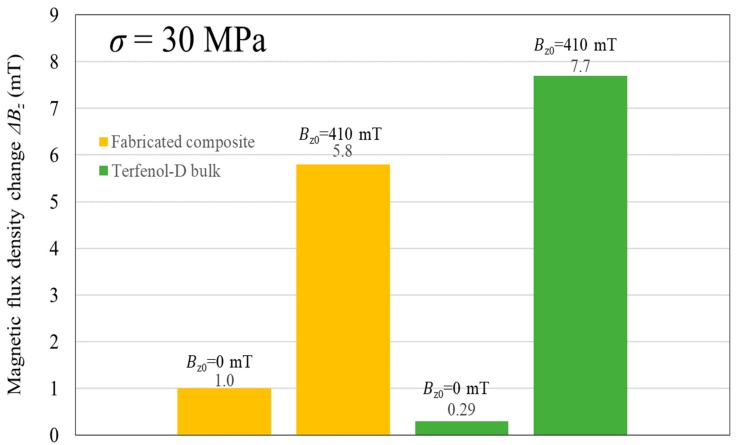
Comparison between fabricated composite and Terfenol-D bulk on the magnetic flux density change at stress *σ* = 30 MPa.

Figure 10 shows the comparison of the magnetic flux density change ∆*B_z_* between the present composite, and Terfenol-D bulk, subjected to compressive stresses of *σ* = 30 MPa. The results of the fabricated composite were 1.0 mT under *Bz*_0_ = 0 mT and 5.8 mT under *Bz*_0_ = 410 mT. It was shown that the magnetic flux density change ∆*B_z_* of the present composite without bias magnetic field was larger than that of Terfenol-D bulk without bias magnetic field. The result of Terfenol-D bulk under *Bz*_0_ = 410 mT showed a large magnetic flux density change. However, Terfenol-D bulk may be broken by repeated compressive stresses due to its brittleness.

## 4. Conclusions

Novel Fe–Co magnetostrictive fiber reinforced plastic composites have been developed here. The Fe–Co fibers were woven into a polyester fabric in order to align them along the same direction and to change the location of the fibers in the composites. The results showed that the magnetic flux density change is related to the initial magnetization of the Fe–Co fiber and the bias magnetic field. Moreover, the magnetic flux density change of the fabricated fiber reinforced plastic composite was larger than that of the plastics with Terfenol-D particles. This work will develop possibilities of lightweight, strong, high performance stress sensors and energy harvesting devices. On the other hand, the development of magnetostrictive sensors and harvesters requires enhancement of magnet and coil qualities. An important challenge in the coming years is a device (e.g., magnet) size and mass reduction. Our future studies will be directed to address these concerns.

## Figures and Tables

**Table 1:** Final dimensions and fabric distribution.

Specimen	Length *l* (mm)	Width *w* (mm)	Thickness *h* (mm)	Fabric Distribution
Type 1	25.05	12.00	7.55	Near the center
Type 2	23.50	11.50	7.55	In one side
